# PLANT PROTEIN BUT NOT ANIMAL PROTEIN CONSUMPTION IS ASSOCIATED WITH FRAILTY THROUGH PLASMA METABOLITES

**DOI:** 10.1093/geroni/igad104.3528

**Published:** 2023-12-21

**Authors:** Tanaka Toshiko, Jayanta Das, Qu Tian, Ruin Moaddel, Ann Zenobia Moore, Katherine Tucker, Sameera Talegawkar, Luigi Ferrucci

**Affiliations:** NIA/IRP/TGB/LSS, Baltimore, Maryland, United States; National Institute on Aging, Baltimore, Maryland, United States; National Institute on Aging, Baltimore, Maryland, United States; National Institute on Aging, Baltimore, Maryland, United States; National Institute on Aging, Baltimore, Maryland, United States; University of Massachusetts at Lowell, Lowell, Massachusetts, United States; The George Washington University, Washington, District of Columbia, United States; National Institute on Aging, Baltimore, Maryland, United States

## Abstract

There is evidence that the association of protein intake and frailty may depend on the source of dietary protein. The mechanism underlying these associations are not clear. In this study, we explore circulating metabolites as mediators of the relationship between dietary protein and of frailty in participants of the Baltimore Longitudinal Study of Aging (BLSA). Cross-sectional analyses in 735 BLSA participants of associations between plant and animal protein intake and frailty. Usual protein intake from plant and animal sources were estimated with a Food Frequency Questionnaire (FFQ) and frailty was assessed with a 44-item Frailty Index (FI). Compared with the lowest quartile, higher quartiles of plant, but not animal, protein were associated with lower FI. Twenty-five plasma metabolites were associated with plant protein intake; of these, 15, including phosphatidylcholines, cholesterol esters, sphingomyelins, and indole metabolites mediated the association between plant protein intake and FI. The protective association between plant protein consumption and FI is mediated by lower abundance of lipid metabolites and higher abundance of tryptophan-related metabolites.

